# Spatial immune niches in gastric cancer immunotherapy resistance: mechanisms and translational implications

**DOI:** 10.3389/fimmu.2026.1905211

**Published:** 2026-08-03

**Authors:** Ziqiang Ling, Renzhe Tang, Botao Yuan, Meng Gao, Yu Li

**Affiliations:** 1 The First Clinical Medical College, Heilongjiang University of Chinese Medicine, Harbin, China; 2 Department of Oncology III, The First Affiliated Hospital, Heilongjiang University of Chinese Medicine, Harbin, China

**Keywords:** cancer-associated fibroblasts, gastric cancer, immunotherapy resistance, predictive biomarkers, spatial transcriptomics, tertiary lymphoid structures, tumor microenvironment, tumor-associated macrophages

## Abstract

Immune checkpoint inhibitors have changed the treatment landscape for gastric and gastroesophageal junction adenocarcinoma, yet durable benefit remains uneven across biomarker-defined groups. PD-L1 combined positive score, MSI/dMMR, tumor mutational burden, EBV status, HER2, and CLDN18.2 guide treatment eligibility, but they do not explain why effector immunity succeeds in one lesion and fails in another. This narrative review uses spatial immune niches as a framework for interpreting immunotherapy resistance in gastric cancer. These niches are tissue compartments in which malignant-cell states, effector-cell access, tertiary lymphoid structures, cancer-associated fibroblasts, tumor-associated macrophages, extracellular matrix, vascular function, and metastatic-site ecology jointly shape response. We synthesize evidence from clinical trials, single-cell and spatial multi-omics, multiplex immunohistochemistry and immunofluorescence, digital pathology, and translational biomarker studies. The review highlights clinically relevant niche states, including lymphoid-organized inflamed, inflamed-but-suppressed, stromal-excluded, myeloid-dominant, vascular/hypoxic, target-antigen-associated, and peritoneal metastatic niches. It further discusses how compact spatial assays could refine patient selection and support niche-matched combination strategies.

## 1 Introduction

Gastric cancer (GC) remains a leading cause of cancer mortality and a therapeutically heterogeneous disease. Immune checkpoint inhibitors (ICIs), particularly PD-1/PD-L1 blockade combined with chemotherapy or targeted agents, have improved outcomes for selected patients with advanced gastric and gastroesophageal junction adenocarcinoma, yet durable benefit remains uneven and primary or acquired resistance is frequent (1–7). Current stratification relies on PD-L1 combined positive score, MSI/dMMR, tumor mutational burden, EBV-associated immune activation, HER2 status, and CLDN18.2 expression. These biomarkers are clinically useful, but they capture only part of the biology that determines whether antitumor immunity can be initiated, sustained, and converted into tumor-cell killing.

A key unresolved issue is that immune responsiveness in GC is not defined solely by the presence or absence of a marker, cell type, or pathway. Similar densities of CD8 T cells, TAMs, CAFs, or TLS-associated lymphocytes can carry different implications depending on their location, contact with tumor cells, separation by stromal, vascular, or metabolic barriers, and the local balance between antigen presentation and checkpoint-mediated suppression (8–11). Immunotherapy resistance should therefore be interpreted not only as a molecular or cellular phenotype, but also as a spatially organized tissue state.

Single-cell sequencing, spatial transcriptomics, multiplex immunohistochemistry/immunofluorescence, digital spatial profiling, and high-plex imaging now make it possible to link cellular composition to tissue architecture in GC and other solid tumors (8, 12–14). These approaches show that tumor nests, invasive fronts, stromal septa, tertiary lymphoid structures, perivascular regions, hypoxic compartments, and metastatic sites can impose distinct immune constraints. Much of this evidence remains exploratory, however, and spatial-omics discoveries should not be treated as clinically validated biomarkers. A structured framework is therefore needed to organize these observations into biologically interpretable and clinically testable categories.

We synthesize evidence that spatial immune organization contributes to GC immunotherapy resistance. We define this concept as a hierarchical framework linking spatial units, cellular niches, and functional immune phenotypes; integrate tumor-intrinsic, stromal, myeloid, metabolic, and temporal mechanisms of resistance; and discuss how spatial biomarkers and emerging technologies could inform biomarker-guided therapeutic strategies. We distinguish hypothesis-generating spatial-omics observations from biomarkers with greater clinical readiness, with the aim of providing a map for future validation rather than presenting spatial niche categories as established clinical tests.

Here, spatial immune niche is used as a hierarchical concept that links anatomical location, local cellular interaction, response-relevant function, and assay feasibility. This framing explains why conventional biomarkers remain necessary but incomplete, and why tumors with similar molecular eligibility can differ in response depending on the tissue context in which effector immunity must operate.

## 2 Definition and conceptual framework of spatial immune niches in gastric cancer

In this Review, spatial immune niche is used as a hierarchical, rather than interchangeable, term. A spatial unit is a physical region defined by tissue architecture, such as tumor nests, invasive fronts, stromal septa, perivascular regions, TLSs, hypoxic areas, and peritoneal metastatic sites (8, 10, 15, 16). A cellular niche is a local microenvironment within such a spatial unit, defined by interacting cell types, matrix organization, and soluble signals, such as a CAF-macrophage niche, a TLS-associated B-cell and T-cell niche, or a perivascular myeloid niche (9, 11, 17). A functional phenotype is the response-relevant tissue state produced by one or more spatial units and cellular niches. We organize these phenotypes into seven working states: lymphoid-organized inflamed, inflamed-but-suppressed, stromal-excluded, myeloid-dominant, vascular/metabolic, target-antigen, and peritoneal metastatic phenotypes.

This hierarchy prevents three concepts from being collapsed into a single label. Anatomical location defines where immune regulation occurs. Cellular-niche composition defines which cells and signals form the local interaction circuit. Functional phenotype defines how these circuits may influence ICI response, resistance, or relapse. Thus, a tumor nest containing proliferating tumor-proximal CD8 T cells represents a different functional state from a stromal septum in which CD8 T cells are retained near collagen-rich CAFs and SPP1-positive macrophages, even when both samples show high total immune-cell density (18–20).

The classic immune-inflamed, immune-excluded, and immune-desert taxonomy remains useful as an initial description, but it is too coarse for GC when used alone (21). In this framework, these labels serve as background descriptors rather than as the final classification system. Inflamed tumors may be lymphoid-organized and potentially ICI-sensitive, or inflamed-but-suppressed when effector cells coexist with Tregs, suppressive TAMs, dysfunctional T cells, and myeloid checkpoints (17, 22–24). Excluded tumors may reflect stromal barriers, myeloid-dominant suppression, vascular dysfunction, hypoxia, metabolic stress, or metastatic-site ecology (9, 25, 26). Desert tumors can reflect low antigenicity, defective antigen presentation, failed dendritic-cell priming, or sampling of a region that missed the invasive front (25, 27–29). The spatial-niche framework therefore refines the classic taxonomy by linking location, cellular interaction, mechanism, and assay-readout in a single interpretive structure.

**Figure 1:**
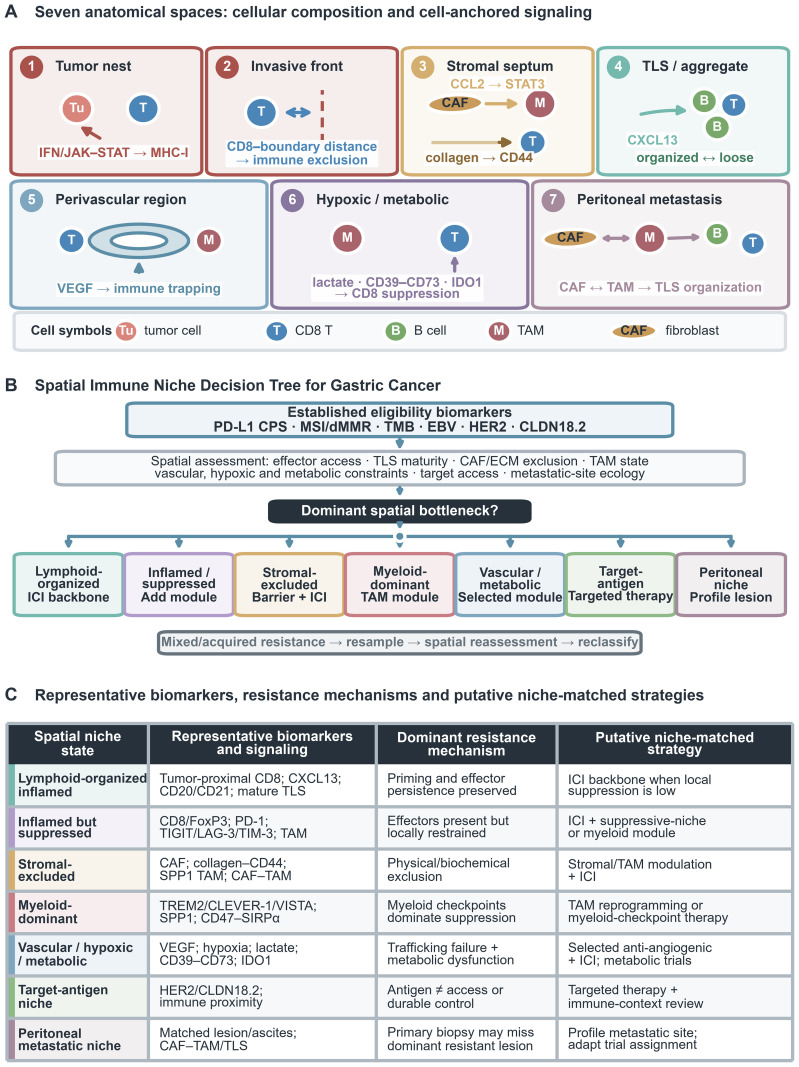
Spatial immune niche decision tree for gastric cancer immunotherapy resistance. **(A)** Seven independent anatomical spaces with representative cellular composition and cell-anchored signaling. The tumor nest links tumor-cell IFN/JAK-STAT signaling to MHC-I; the invasive front uses CD8-to-boundary distance to represent immune exclusion; the stromal septum shows CAF-to-TAM CCL2-STAT3 signaling and collagen-CD44 interactions; the tertiary lymphoid structure (TLS)/lymphocyte aggregate shows CXCL13-associated lymphoid organization; the perivascular region links VEGF to abnormal vessels and immune trapping; the hypoxic/metabolic area links lactate, CD39-CD73 and IDO1 to local CD8 T-cell suppression; and the peritoneal metastatic site shows reciprocal CAF-TAM interactions and site-specific TLS organization. A single-headed arrow denotes signaling or functional influence; a double-headed arrow denotes spatial distance or reciprocal interaction. Tu, tumor cell; T, CD8 T cell; B, B cell; M, macrophage; CAF, cancer-associated fibroblast. **(B)** Spatial immune-niche decision tree. Downward arrows indicate the sequential assessment of established eligibility biomarkers, spatial features and the dominant spatial bottleneck. The horizontal branch and seven separate arrowheads indicate alternative, potentially coexisting functional niche classifications rather than a temporal sequence. Mixed or acquired resistance prompts repeat sampling, spatial reassessment and niche reclassification. Established eligibility biomarkers include PD-L1 combined positive score (CPS), microsatellite instability/mismatch-repair deficiency (MSI/dMMR), tumor mutational burden (TMB), Epstein-Barr virus (EBV), HER2 and CLDN18.2. **(C)** Representative niche-associated biomarkers and signaling programs, dominant resistance mechanisms and putative niche-matched therapeutic strategies. The proposed classes and therapeutic assignments are conceptual hypotheses derived from the reviewed evidence; multiple classes may coexist, and prospective validation is required before treatment assignment. ICI, immune-checkpoint inhibitor; ECM, extracellular matrix; TAM, tumor-associated macrophage.

To operationalize this framework, we propose a spatial immune-niche decision tree that integrates anatomical location, dominant cellular neighborhoods, candidate biomarkers and signaling programs, resistance mechanisms, and putative niche-matched therapeutic strategies (**Figure 1**).

## 3 Technologies for mapping spatial immunity

Multiplex IHC and multiplex immunofluorescence are the most deployable spatial assays because they preserve architecture, work on FFPE tissue, and quantify cell identity, proximity, and colocalization. In gastric cancer, these methods have been used to assess PD-1-positive CD8 cells, FoxP3-positive cells, HER2-associated immune remodeling, TLS composition, and pretreatment features linked to immunochemotherapy response (30–34).

Spatial transcriptomics and single-cell RNA sequencing are discovery engines rather than ready-made clinical tests. They identify lymphocyte-aggregated regions, stromal danger zones, CAF-macrophage crosstalk, peritoneal ecosystems, and cell-state programs. However, spot resolution, deconvolution, cohort size, and validation remain limiting (8, 9, 15, 16, 35–37).

Digital spatial profiling bridges discovery and clinical translation by measuring targeted programs in annotated tumor, stromal, invasive-front, or immune-rich regions. Its biopsy compatibility is attractive, but the selected region of interest can determine the conclusion (32).

Digital pathology and computational spatial statistics are needed to make spatial findings reproducible. Clinically usable biomarkers will likely combine compact staining panels with interpretable measurements of neighborhoods, distances, gradients, and cell-state proximity (13).

Beyond mIHC/mIF, high-plex imaging platforms are becoming increasingly important in spatial oncology. Imaging mass cytometry (IMC), co-detection by indexing (CODEX), and multiplex ion beam imaging (MIBI) can profile dozens of markers while preserving tissue architecture (14, 38, 39). Their value lies in measuring cell identity, functional state, and neighborhood organization in the same section. This is particularly relevant to GC, in which a single lesion may contain tumor nests with effector access, stromal regions with immune exclusion, TLS-like aggregates, vascular or hypoxic compartments, and metastatic-site-specific immune programs.

These high-dimensional platforms are best regarded as discovery and validation tools rather than immediate routine diagnostics. They can identify candidate neighborhoods, marker combinations, and decision rules, but clinical translation will usually require reduction to FFPE-compatible panels, digital pathology scores, or targeted spatial profiling assays. AI-enabled pathology can support this transition by quantifying TLS maturity, tumor-stroma boundaries, cell distances, and regional heterogeneity at a scale that is difficult to reproduce manually. Digital TLS and multimodality studies in GC support this direction, although implementation will require external validation, assay standardization, and clinically interpretable thresholds (40, 41).

For translation, spatial evidence can be tiered into discovery atlases, validation cohorts, and deployment-ready assays. Atlas studies generate hypotheses, whereas FFPE-compatible multiplex panels, digital TLS scoring, PD-L1 assay-comparison work, pathology-AI models, and paired treatment cohorts move the field closer to clinical use (30–32, 40–43).

## 4 Spatial distribution of effector immune cells

### 4.1 CD8 T-cell access and functional state

CD8 T cells are central to ICI response, but their spatial distribution is more informative than their absolute density. A tumor with intratumoral CD8 cells in direct proximity to malignant cells is biologically distinct from one in which CD8 cells accumulate in stromal zones. Intratumoral CD8 cells have been proposed as a positive predictor of chemoimmunotherapy response even in PD-L1-negative advanced gastric cancer, supporting the idea that effector-cell localization can add information beyond CPS (44).

PD-1-positive CD8 T cells require nuanced interpretation. They may represent an exhausted population, but they can also indicate antigen experience and potential reinvigoration by PD-1 blockade. Multiplex studies of gastric cancer suggest that PD-1-positive CD8 cells have clinical relevance, particularly when evaluated with markers of proliferation, cytotoxicity, and immune context (30, 31). A spatially informed analysis should therefore distinguish proliferative tumor-proximal PD-1-positive CD8 cells from dysfunctional CD8 cells trapped in suppressive niches.

The relationship between CD8 T cells and suppressive cells is equally important. CD8 cells surrounded by Tregs, SPP1-positive macrophages, or CAF-derived ECM may be unable to execute cytotoxicity even when numerically abundant. Integrated immune-resistance studies linking exhausted CD8 T cells to macrophage- and stromal-associated barriers support this view (18, 45). Thus, CD8 localization should be paired with neighborhood analysis rather than reported only as bulk density.

### 4.2 B cells and tertiary lymphoid structures as immune-organizing niches

TLSs provide one of the clearest examples of this compartmental logic. Rather than merely forming lymphocyte accumulations, they can act as local immune-organizing structures that support antigen presentation, B-cell maturation, chemokine gradients, and T-cell activation. Single-cell and spatial transcriptomic studies indicate that TLS density, maturation, and localization have prognostic and immunotherapeutic relevance in gastric cancer (17, 46).

Mechanistically, TLS-associated B cells may enhance CD8 tissue-resident memory-like responses to PD-1 blockade, and CXCL13-positive CD8 T-cell circuits may promote TLS formation and immunotherapy efficacy (22, 47). These findings suggest that B cells are not only antibody-producing bystanders. In the right spatial context, they may help maintain local antigen presentation, recruit effector cells, and sustain productive immune memory.

TLSs should not, however, be treated as universally favorable. Their impact may differ by maturity, location, cellular composition, and metastatic setting. Mapping of primary and peritoneal metastatic gastric cancer shows that TLS complexity and diversity vary across sites (10). A mature TLS containing organized B-cell and T-cell zones may indicate local immune competence, whereas a loose lymphoid aggregate in a suppressive metastatic niche may have different implications.

Recent validation-oriented TLS studies strengthen the translational argument. A multicenter digital-pathology assessment linked TLS features with therapeutic response, while artificial-intelligence and multimodality approaches are beginning to separate TLS maturity from simple lymphocyte aggregation (40, 41). Preoperative-treatment cohorts also suggest that the balance among PD-1-positive Tregs, PD-1-positive CD8 cells, and TLSs may influence response (23). Therefore, the most useful TLS biomarker is unlikely to be presence versus absence. It should report maturity, location, cellular balance, and proximity to tumor nests.

TLS interpretation should avoid a binary favorable-versus-unfavorable framing. Mature TLSs imply organized B-cell and T-cell areas, follicular dendritic-cell-like support, germinal-center activity or maturation features, and proximity to tumor regions where antigen encounter and effector recruitment can occur. Immature TLSs or loose lymphoid aggregates may instead represent incomplete organization or an inflammatory byproduct (17, 22, 40, 41, 46, 47). In gastric cancer, this distinction is especially important because primary tumors and peritoneal metastases may contain different lymphoid architectures. A TLS-like aggregate embedded in a CAF-macrophage-rich metastatic niche may not carry the same implication as a mature invasive-front TLS (10, 23, 48).

### 4.3 Antigen presentation, dendritic cells, and underdeveloped effector compartments

Compared with CD8 cells, B cells, and TLSs, the supplied literature base is less developed for dendritic-cell spatial biology, NK-cell localization, and rare effector T-cell subsets in gastric cancer. This is a meaningful gap. Effective ICI response requires antigen presentation, priming, trafficking, and effector function. If dendritic-cell maturation or cross-presentation is defective, the tumor may remain immune desert even when tumor antigens exist.

Future spatial panels should incorporate dendritic-cell maturation markers, antigen-presentation machinery, NK-cell activation, and additional inhibitory receptors. Without these markers, immune-desert and immune-excluded phenotypes may be confused. A tumor may appear cold because effector cells never entered, because dendritic-cell priming failed, or because sampling missed the relevant immune compartment. These states require different interventions.

### 4.4 Tumor-intrinsic resistance within spatial immune niches

Spatial resistance is not generated solely by immune and stromal cells. Tumor-intrinsic alterations can determine whether an otherwise immune-rich niche remains visible to cytotoxic lymphocytes. Defects in antigen processing and presentation, including MHC-I downregulation, β2-microglobulin loss, impaired antigen loading, or reduced interferon-responsive presentation programs, may weaken tumor-cell recognition even when CD8 T cells reach the tumor nest. In this setting, spatial assays may detect tumor-proximal lymphocytes, but the local interaction fails because malignant cells no longer present sufficient antigenic targets (27, 28, 49, 50).

Interferon signaling abnormalities provide a related but distinct route to resistance. Productive interferon-gamma signaling can increase antigen presentation, chemokine expression, and local immune recruitment. By contrast, disrupted JAK-STAT signaling or chronic interferon exposure can produce immune invisibility or adaptive resistance, depending on timing and cellular context. Spatial profiling is useful because it can distinguish tumor-intrinsic presentation defects from extrinsic access barriers. A CD8-poor tumor nest adjacent to immune-rich stroma suggests exclusion, whereas a CD8-rich tumor nest with weak antigen-presentation machinery suggests a tumor-cell visibility defect.

These mechanisms should be integrated with established GC biomarkers rather than treated as separate categories. MSI/dMMR and EBV-associated tumors may be immunogenic, but response still depends on intact antigen presentation, dendritic-cell support, effector-cell entry, and limited local suppression. Conversely, chromosomally unstable or target-antigen-positive tumors may retain spatial immune competence in selected regions despite lower global immunogenicity. A practical spatial panel should therefore measure not only immune-cell position, but also tumor-cell antigen-presentation state, ideally through compact markers linked to MHC-I expression, interferon-response programs, and effector-cell proximity.

## 5 Immunosuppressive spatial niches

### 5.1 CAF-macrophage crosstalk

The most prominent suppressive pattern in the local evidence base is the convergence of CAFs, macrophages, and ECM. Spatial dissection of gastric cancer has identified immunosuppressive crosstalk between CCL2-positive fibroblasts and STAT3-activated macrophages (9). This is a strong example of a spatially interpretable resistance mechanism: fibroblasts and macrophages are not merely present; they are organized into interacting neighborhoods that can shape immune function.

Additional studies reinforce this model. In H. pylori-associated gastric cancer, CAF-mediated immune modulation has been mapped spatially and functionally (51). Multi-omics analyses have highlighted interactions between GREM1-positive fibroblasts and SPP1-positive macrophages (52). These studies converge on the idea that CAFs and TAMs can form cooperative suppressive circuits that recruit each other, remodel ECM, and limit cytotoxic T-cell function.

The therapeutic implication is that targeting only PD-1 may be insufficient when the dominant barrier is stromal-myeloid. If effector cells cannot enter tumor nests or remain functional after entry, checkpoint blockade may have limited impact. In these tumors, stromal remodeling, macrophage reprogramming, chemokine modulation, or vascular normalization may be needed before T-cell reinvigoration becomes effective.

### 5.2 ECM, fibrosis, and immune exclusion

ECM is not an inert scaffold. Collagen architecture, stiffness, ligand availability, and stromal density can determine whether immune cells move toward tumor cells or remain confined to stromal regions. The collagen-CD44 axis and tumor-associated fibrosis have been linked to a poor-prognosis subtype and immunosuppression in gastric cancer (19). These findings support a model in which ECM remodeling creates both physical and biochemical resistance.

SUSD2-positive CAFs and other CAF subtypes further illustrate why stromal biology cannot be reduced to total fibroblast abundance (53). Some CAF programs may be strongly immunosuppressive, whereas others may have neutral or even immune-supportive functions depending on disease stage and location. Spatial assays are needed to distinguish CAF subtypes at the invasive front, tumor core, perivascular areas, and post-treatment residual lesions.

The immune-excluded phenotype can therefore be interpreted as an active tissue state. CAF-driven collagen-integrin signaling, MIF signaling, SPP1-positive macrophages, and CCDC80-positive danger-zone programs all contribute to spatial resistance (15, 18, 45). These signals do not simply correlate with resistance; they offer mechanistic hypotheses for why effector cells fail to penetrate tumor nests or remain functional after entry.

### 5.3 TAM heterogeneity and myeloid checkpoints

TAMs are among the most plastic and clinically important immune cells in gastric cancer. TREM2-positive TAMs, VISTA-high myeloid landscapes, and CLEVER-1-positive macrophage programs have emerged as suppressive axes that may limit PD-1 response (11, 20, 54). These observations are consistent with a myeloid-dominant resistance niche.

Myeloid-dominant resistance differs from classic T-cell exhaustion. In a T-cell exhaustion model, PD-1 blockade can restore function if T cells retain reinvigoration potential. In a myeloid-dominant model, macrophages and related cells maintain suppressive cytokines, inhibitory ligands, phagocytic checkpoints, metabolic stress, and ECM remodeling. PD-1 blockade alone may not remove these constraints.

This distinction matters for combination therapy. CLEVER-1 blockade has been reported to restrain immune evasion and enhance anti-PD-1 immunotherapy in gastric cancer (20). TREM2-positive TAMs and VISTA-high landscapes suggest other myeloid-directed strategies (11, 54). Blood TCTP may also reflect systemic immunosuppressive features and poor outcomes in metastatic gastric cancer (55). Tissue and blood markers may eventually be combined to identify patients with macrophage-dominant resistance.

### 5.4 Vascular, hypoxic, and peritoneal metastatic niches

Vascular and hypoxic niches can prevent immune entry even when effector cells exist systemically. Abnormal vessels impair trafficking, hypoxia promotes suppressive myeloid polarization, and perivascular ECM can trap lymphocytes. Anti-angiogenic therapy may therefore have immune-modulatory effects, but those effects are not uniform. Spatial transcriptomics and plasma proteomics have identified microenvironmental features associated with primary resistance to anti-PD-1/PD-L1 plus antiangiogenesis (26).

The ramucirumab plus pembrolizumab experience further suggests that benefit may depend on spatially resolved TME trajectories rather than a single baseline marker (56). This is important because vascular normalization can be transient and context dependent. A tumor may initially improve immune access but later develop compensatory stromal or myeloid barriers.

This biological rationale has been tested through several clinical backbones. REGARD and RAINBOW established VEGFR2 inhibition with ramucirumab in previously treated gastric cancer, providing the anti-angiogenic foundation for later immunotherapy combinations (57, 58). Retrospective anti-PD-1 plus anti-angiogenesis plus chemotherapy data add real-world context for HER2-negative advanced disease (59). REGONIVO, LEAP-015, and updated multikinase inhibitor plus PD-1 data suggest that vascular targeting can help selected tumors but does not automatically overcome resistance (60–62). Spatial resistance studies are therefore important because they explain why some vascular-normalization strategies fail when stromal or myeloid suppression persists.

Peritoneal metastasis adds another layer. Spatially resolved analysis of gastric cancer peritoneal metastases shows that metastatic organotropism is associated with specific tumor and microenvironmental alterations (16). Peritoneal disease should not be assumed to share the same immune architecture as the primary tumor. Sampling strategies and spatial biomarker panels should therefore include metastatic sites whenever clinically feasible.

Newer peritoneal-metastasis studies make this point more clinically concrete. CAF-macrophage crosstalk in gastric cancer peritoneal metastases can govern immune-checkpoint blockade response, and genomic and immune microenvironment features have been linked to chemoimmunotherapy response in peritoneal disease (48, 63). These data support treating peritoneal metastasis as a distinct immune niche, not merely as an anatomic site of advanced tumor burden.

### 5.5 Metabolic immune suppression in spatial niches

Metabolic stress can convert an immune-infiltrated region into a functionally suppressed niche. Hypoxia is the most spatially intuitive example because it colocalizes with abnormal vessels, poor perfusion, and immune-excluded regions. Hypoxic areas can favor suppressive myeloid polarization, impair effector-cell function, and reinforce stromal remodeling. In GC, this process should be interpreted alongside vascular and perivascular architecture rather than as a diffuse tumor-wide property (25, 26, 56, 64).

Lactate accumulation, adenosine signaling, and tryptophan catabolism through the IDO1 pathway provide additional routes through which local metabolism can suppress antitumor immunity. Lactate-rich regions can impair cytotoxic T-cell and NK-cell function and support suppressive macrophage states (65). The CD39-CD73-adenosine axis can dampen effector activity through adenosine-receptor signaling, whereas IDO1-mediated tryptophan depletion and kynurenine accumulation can promote T-cell dysfunction and regulatory immune programs (66, 67).

These pathways strengthen the spatial-niche model because metabolic constraints are likely to operate as gradients. A tumor may contain immune-competent regions adjacent to metabolically hostile regions, and a single biopsy may overestimate or underestimate the dominant barrier. For clinical translation, metabolic suppression should be measured together with cell position, vascular state, macrophage programs, and effector-cell function. Such integration would help distinguish an inflamed-but-suppressed niche from a stromal-excluded or myeloid-dominant niche when morphology alone is ambiguous.

## 6 Spatial immune phenotypes and ICI response

Spatial immune phenotypes should be interpreted as response programs rather than static labels. An inflamed/TLS-rich phenotype is most likely to benefit from ICIs when tumor-proximal CD8 cells, CXCL13-rich circuits, B-cell support, and mature TLS architecture are present (8, 17, 22, 46, 47). Such tumors may already contain the ingredients required for checkpoint reinvigoration. The therapeutic question is whether these ingredients are functionally intact and not overwhelmed by local suppression.

An inflamed-but-suppressed phenotype is different. These tumors may contain abundant immune cells but also high Treg, TAM, VISTA, CLEVER-1, or ECM-associated suppression. They may show partial response, mixed response, or early acquired resistance. In this setting, adding another T-cell checkpoint may not be enough; the relevant target may be the suppressive neighborhood.

A stromal-excluded phenotype is characterized by immune-cell accumulation outside tumor nests. In gastric cancer, the supporting evidence points to CAF-rich stroma, collagen remodeling, SPP1-positive macrophages, CCDC80-positive fibroblast programs, SUSD2-positive CAFs, and danger-zone architecture (15, 18, 45, 53). Such tumors may be candidates for stromal remodeling, macrophage reprogramming, anti-angiogenic normalization, or chemokine modulation combined with ICI therapy.

A myeloid-dominant phenotype contains suppressive macrophage programs that may operate independently of classical T-cell exhaustion. TREM2-positive TAMs, CLEVER-1-positive TAMs, VISTA-high myeloid landscapes, and TCTP-associated immune suppression all support this category (11, 20, 54, 55). The presence of macrophage-dominant PD-L1 signal may also explain why CPS can be clinically ambiguous.

For practical use, inflamed-but-suppressed and myeloid-dominant phenotypes should be defined by operational criteria rather than by a single marker. An inflamed-but-suppressed phenotype contains measurable immune infiltration, but effector function is constrained by local suppressive signals. It can be provisionally recognized when tumor-proximal or stromal CD8 T cells coexist with Tregs, inhibitory receptors, suppressive TAMs, or ECM-rich neighborhoods. T-cell functional markers can help distinguish a potentially reinvigoratable state from a deeply dysfunctional state. Proliferation markers such as Ki-67, cytotoxic molecules such as granzyme B (GZMB), and exhaustion-associated markers such as TIGIT, LAG-3, TIM-3, or PD-1 should be interpreted together with tumor-cell proximity and local suppressive context (24, 31, 68–70).

A myeloid-dominant phenotype should be used when macrophage and related myeloid programs appear to organize the dominant suppressive circuit. This phenotype may include TREM2-positive TAMs, CLEVER-1-positive TAMs, VISTA-high myeloid states, SPP1-positive macrophages, or CAF-macrophage crosstalk. T cells may be present, but they are unlikely to be the only therapeutic bottleneck. The therapeutic hypothesis therefore differs from a simple T-cell exhaustion model: myeloid reprogramming, CD47-SIRPα blockade, VISTA-directed strategies, or TAM-targeted combinations may be more rational than adding another T-cell checkpoint alone (11, 71–74). This classification should be presented as a working framework that awaits prospective validation, not as an established clinical algorithm.

An immune-desert phenotype should be subdivided rather than treated as a single group. One desert tumor may be antigen-poor, another may have defective antigen presentation, another may have immune exclusion outside the sampled region, and another may be shaped by systemic immune suppression. Single-cell profiling of gastric signet-ring cell carcinoma also illustrates how histologic contexts can carry immune irresponsiveness that is not captured by PD-L1 alone (75). MSI-H, dMMR, and EBV-associated studies show that immunogenic molecular categories still require immune-context interpretation (76–79).

Clinical response studies increasingly support this spatial framework. Spatial features associated with anti-PD-1/PD-L1 plus antiangiogenesis resistance, ramucirumab plus pembrolizumab benefit, neoadjuvant immunochemotherapy response, and stromal-immune crosstalk signatures all suggest that response is a property of tissue neighborhoods and not only a tumor-cell molecular state (26, 32, 56, 80, 81).

Treatment-paired multiomics adds a dynamic dimension to this framework. In locally advanced gastric or gastroesophageal junction cancer, neoadjuvant immunotherapy plus chemotherapy can remodel the tumor microenvironment. Immune-signature studies in first-line anti-PD-1 settings also suggest that response-associated features may differ from chemotherapy-associated features (42, 82). Spatial phenotyping should therefore be designed for baseline prediction and on-treatment reinterpretation, especially when mixed response or acquired resistance emerges.

## 7 Spatial biomarkers for precision stratification

A clinically useful spatial biomarker strategy should be layered. The first layer is the established molecular and immunohistochemical framework: PD-L1 CPS, MSI/dMMR, TMB, EBV status, HER2, and CLDN18.2 (83). These markers remain essential because they guide approved therapies and trial eligibility. However, they should be interpreted as entry points rather than complete predictors. For PD-L1 specifically, assay and scoring variability remains a practical barrier. Comparative work across 28-8, 22C3, and SP263 assays and across CPS and tumor-area positivity emphasizes that PD-L1 is not a single stable measurement (43). Dynamic PD-L1 imaging and immunoPET-oriented studies further support the idea that PD-L1 expression is heterogeneous across lesions and over time (84). Spatial biomarkers should therefore complement PD-L1 rather than serve as decorative additions to a fixed score.

The second layer should measure effector access. This includes intratumoral CD8 localization, PD-1-positive CD8 state, granzyme or proliferation markers, tumor-cell proximity, and exclusion distance. CD8 cells in tumor nests are biologically different from CD8 cells in distant stroma (30, 31, 44). A spatial CD8 score should therefore combine density with distance and functional markers.

The third layer should measure lymphoid organization. TLS maturity, B-cell/T-cell pairing, CXCL13-positive T cells, lymphocyte-aggregated regions, and germinal-center-like features may indicate local immune priming and memory support (8, 17, 22, 46, 47). TLS biomarkers should specify location and maturity because primary tumors and peritoneal metastases may contain different lymphoid structures (10). Digital TLS scoring is one of the most near-term opportunities for clinical translation. It can be built from routine histology or compact multiplex panels and then validated against response and survival endpoints (40, 41). The key requirement is to distinguish mature, organized TLSs from loose lymphocyte aggregates and to report whether the lymphoid structure is tumor-proximal, stromal, invasive-front, or metastatic-site associated.

The fourth layer should measure stromal and myeloid suppression. CAF/ECM burden, collagen-CD44 signaling, SUSD2-positive or CCDC80-positive fibroblast programs, SPP1-positive macrophages, TREM2-positive TAMs, CLEVER-1-positive TAMs, VISTA-high myeloid states, and TCTP-associated systemic suppression all represent candidate resistance readouts (11, 15, 19, 20, 52–55). These features can help separate inflamed-but-suppressed tumors from truly inflamed-sensitive tumors.

The fifth layer should measure vascular and metastatic context. Angiogenic, hypoxic, and perivascular signatures may identify tumors that require vascular normalization before efficient immune entry (26, 56). Peritoneal metastasis should be treated as a distinct sampling and biomarker problem rather than an extension of the primary tumor (16). For anti-angiogenic combinations, the biomarker should also determine whether vessels, hypoxia, macrophages, and CAF/ECM programs are aligned toward immune entry or immune trapping. A patient selected only by PD-L1 positivity may still resist anti-PD-1 plus antiangiogenesis if the dominant spatial pattern is stromal-myeloid exclusion rather than reversible vascular dysfunction (26, 56, 59–62).

A practical composite biomarker could classify tumors into seven actionable niche states: lymphoid-organized inflamed, inflamed-but-suppressed, stromal-excluded, myeloid-dominant, vascular/metabolic, target-antigen-associated, and peritoneal metastatic niches. This classification would not replace existing biomarkers. Instead, it would serve as an overlay that generates testable explanations for divergent responses, pending prospective validation **Table 1**.

**Table 1:** Actionable spatial immune-niche states in gastric cancer immunotherapy resistance.

Niche state	Spatial pattern	Representative evidence	Key markers/readouts	Resistance mechanism	Therapeutic hypothesis
Lymphoid-organized inflamed	Tumor-proximal CD8 cells, mature TLSs, CXCL13/B-cell circuits	(8, 17, 22, 46, 47)	CD8 distance; CXCL13/CD20/CD21; TLS maturity	Local priming and effector persistence are preserved.	ICI backbone; avoid unnecessary escalation when suppression is low.
Inflamed-but-suppressed	Immune-rich neighborhoods with Tregs, exhausted T cells, or inhibitory TAM states	(11, 20, 23, 30, 31, 54, 55)	CD8/FoxP3 ratio; PD-1 state; VISTA/CLEVER-1/TAM panel	Immune cells are present but locally restrained.	Add suppressive-niche or myeloid-directed strategy.
Stromal-excluded	CD8 cells retained in CAF/ECM-rich stroma or invasive-front barriers	(9, 15, 18, 19, 45, 53)	CD8-to-tumor distance; collagen/CAF markers; CCDC80/SUSD2/SPP1	Physical and biochemical exclusion prevents tumor-cell contact.	Consider stromal remodeling, macrophage reprogramming, or chemokine modulation.
Myeloid-dominant	TAM-rich neighborhoods with SPP1, TREM2, VISTA, CLEVER-1, or CAF-TAM crosstalk	(11, 20, 48, 54, 55)	TAM-state panel; myeloid-checkpoint markers; TAM-CD8 proximity	Macrophage programs dominate suppression independent of classic T-cell exhaustion.	Prioritize TAM reprogramming or myeloid-checkpoint combinations.
vascular/metabolic-excluded	Abnormal vessels, hypoxia, perivascular trapping, and antiangiogenic-resistance programs	(26, 56–62)	Hypoxia/VEGF markers; perivascular CD8 localization; plasma proteomics	Vascular dysfunction limits entry; stromal-myeloid escape may persist.	Use antiangiogenic combinations with spatial selection.
Target-antigen niche	HER2 or CLDN18.2 heterogeneity embedded in distinct immune/stromal contexts	(91, 92, 94–98)	Target IHC plus immune proximity; post-treatment sampling	Target positivity does not ensure immune accessibility or durable control.	Match HER2/CLDN18.2 therapy with immune-context and resistance reassessment.
Metastatic-site niche	Peritoneal or other metastatic ecosystems with site-specific CAF/TAM and lymphoid architecture	(10, 16, 48, 80)	Matched primary-metastasis profiling; peritoneal lesion/ascites sampling	Primary-tumor scores may miss the dominant resistant lesion.	Profile metastatic sites when feasible and adapt trial assignment.

CAF, cancer-associated fibroblast; ECM, extracellular matrix; ICI, immune checkpoint inhibitor; TAM, tumor-associated macrophage; TLS, tertiary lymphoid structure.

## 8 From discovery to clinical testing

Spatial biomarkers should be staged according to evidentiary maturity. At the discovery level, spatial transcriptomics, single-cell multi-omics, high-plex imaging, and computational neighborhood analysis identify candidate niches and mechanisms. These studies are valuable because they reveal unexpected cell circuits, region-specific programs, and metastatic-site differences, but they are often constrained by cohort size, sampling design, platform resolution, and retrospective association.

At the validation level, candidate readouts must be tested in independent cohorts with predefined definitions. TLS maturity, intratumoral CD8 localization, CD8-to-tumor distance, the balance between PD-1-positive CD8 cells and Tregs, CAF/ECM exclusion, TAM-state panels, and vascular/metabolic features are plausible candidates because they can be measured in FFPE tissue or compact multiplex panels. Among these, TLS maturity and digital pathology-based lymphoid organization may be closest to clinical testing because they can be derived from routine histology, immunohistochemistry, or small multiplex panels and have emerging GC validation evidence (40, 41). CD8-to-tumor distance and tumor-proximal effector-state scoring are also attractive because they measure the physical requirement for tumor-cell killing (30, 31, 44).

At the deployment level, a biomarker must inform a treatment decision rather than merely predict prognosis. A deployable spatial assay should answer a clinically actionable question: does the tumor contain a lymphoid-organized inflamed phenotype suitable for ICI-based therapy, a stromal-excluded phenotype requiring barrier modulation, a myeloid-dominant phenotype requiring TAM or myeloid-checkpoint targeting, a vascular/metabolic phenotype suitable for vascular-normalization or metabolic-axis strategies, or a target-antigen niche in which HER2 or CLDN18.2 expression is spatially inaccessible? Until prospective treatment-assignment trials address these questions, niche-matched therapy should be framed as a translational hypothesis rather than as a validated clinical standard.

## 9 Therapeutic implications

Therapeutic implications should be organized around testable niche-matched decisions, not around an additive list of combinations. **Table 2** anchors those decisions in the clinical and translational literature. It separates established treatment backbones from spatial evidence that explains when those backbones may fail.

**Table 2:** Clinical and translational evidence anchors supporting the spatial-niche thesis.

Evidence domain	Representative studies	Representative evidence	Main implication	Spatial interpretation/limitation
First-line PD-1 plus chemotherapy	CheckMate 649, ATTRACTION-4, KEYNOTE-859, RATIONALE-305, ORIENT-16, KEYNOTE-062, MSI-H pooled analyses	(1–7)	Benefit is established but heterogeneous across biomarkers, geography, backbone, and endpoints.	Trial positivity does not specify the resistant tissue architecture.
HER2-positive disease	ToGA, KEYNOTE-811, HER2 TME remodeling, DESTINY-Gastric biomarker/resistance studies	(33, 34, 87–92, 98)	HER2 should be treated as a target-antigen immune ecology, not only as a binary target.	Post-antibody or ADC resistance may be spatially and temporally heterogeneous.
CLDN18.2-positive disease	FAST, SPOTLIGHT, GLOW, ILUSTRO, CLDN18.2 mIHC	(93–97)	A new epithelial target raises immediate questions of immune access and stromal exclusion.	Antigen expression does not equal effector accessibility.
TLS and spatial validation	Spatial TLS studies, digital TLS scoring, AI TLS maturity, PD-1+ Treg/CD8 balance	(10, 17, 22, 23, 40, 41, 46, 47)	TLS maturity and location are among the most deployable spatial biomarkers.	Loose lymphoid aggregates should not be overinterpreted as mature TLSs.
Antiangiogenic combinations	REGARD, RAINBOW, REGONIVO, LEAP-015, updated multikinase+PD-1 data, spatial resistance studies	(26, 56–62)	Rationale is strong but benefit is inconsistent without spatial selection.	CAF/TAM-dominant tumors may not respond to vascular normalization alone.
Spatial evolution and metastasis	Spatial ecosystem evolution, neoadjuvant remodeling, peritoneal CAF-TAM response studies	(10, 12, 16, 42, 48, 63)	Resistance is dynamic and site-specific rather than fixed at baseline.	Primary biopsies may not represent peritoneal or post-treatment resistant lesions.

ADC, antibody-drug conjugate; AI, artificial intelligence; mIHC, multiplex immunohistochemistry; PD-1, programmed cell death protein 1; TME, tumor microenvironment.

The main therapeutic implication is that combination therapy should be niche-matched. ICI plus chemotherapy remains a major backbone, and neoadjuvant studies show that immune features before and after treatment can be associated with response (32, 80, 81). Chemotherapy may increase antigen release and alter immune composition, but it will not necessarily overcome a dense CAF/ECM barrier or macrophage-dominant suppression **Table 2**.

ICI plus anti-angiogenic therapy is biologically rational because vascular normalization can improve immune trafficking and reduce hypoxia-associated suppression. However, spatial and proteomic studies show that primary resistance to anti-PD-1/PD-L1 plus antiangiogenesis still occurs when suppressive microenvironmental programs dominate (26). Ramucirumab plus pembrolizumab data also suggest that spatial TME trajectories may better define benefit than static markers alone (56).

A practical translational interpretation is that anti-angiogenic therapy should be tested preferentially in tumors with a vascular/metabolic spatial phenotype rather than used as a generic enhancer of ICI. Trials and cohorts support both sides of the argument: early signals with regorafenib or lenvatinib plus PD-1 blockade indicate that vascular and myeloid remodeling can be therapeutically relevant, whereas phase 3 and updated datasets highlight limits when resistant tissue programs are not selected (60–62). Spatial transcriptomics and plasma proteomics can bridge these clinical outcomes with mechanism (26, 56, 59).

A practical screening proposal follows from this balance. Before assigning anti-PD-1 plus antiangiogenesis, a compact spatial panel could determine whether the main barrier is reversible vascular dysfunction, stromal exclusion, myeloid dominance, or metastatic-site ecology. This would test whether antiangiogenic combinations can move from empiric escalation toward niche-selected therapy.

Stromal modulation is particularly relevant for immune-excluded gastric cancer. Collagen-CD44 targeting, CAF phenotype modulation, and disruption of fibroblast-macrophage crosstalk all align with the spatial exclusion model (9, 19, 45, 51). The danger is that CAFs are heterogeneous. A broad anti-stromal strategy could remove suppressive barriers but might also disrupt immune-supportive architecture. Patient selection by spatial phenotype will therefore be critical.

Myeloid reprogramming is a second major route. If resistance is driven by TREM2-positive, CLEVER-1-positive, VISTA-high, SPP1-positive, or related macrophage states, therapies that reprogram TAMs or block myeloid checkpoints may be more rational than adding another T-cell checkpoint (11, 20, 54, 55). Tissue spatial markers and blood markers such as TCTP may eventually help identify this subgroup (55).

Emerging immunotherapeutic targets should be interpreted through the same spatial logic. TIGIT, LAG-3, and TIM-3 primarily refine the interpretation of T-cell dysfunction and checkpoint redundancy. Their presence is most informative when evaluated together with T-cell location, proliferation, cytotoxic markers, and suppressive neighborhood context. A TIGIT-positive or LAG-3-positive CD8 T cell inside a tumor nest may imply a different therapeutic opportunity from the same marker expressed by T cells retained in CAF-rich stroma or surrounded by suppressive TAMs (24, 69, 70, 85, 86).

Myeloid and innate immune targets may be particularly relevant in myeloid-dominant niches. CD47-SIRPα blockade could be considered when macrophage-mediated phagocytic checkpoints and tumor-cell do-not-eat-me signals are prominent. NKG2A-directed strategies may be relevant when NK-cell or cytotoxic T-cell inhibition contributes to local immune paralysis. These approaches should not be presented as universally applicable add-ons to PD-1 blockade. They should be linked to spatial evidence identifying the dominant suppressive compartment, such as TAM-rich neighborhoods, myeloid-checkpoint expression, NK/T-cell exclusion, or absence of reinvigoratable tumor-proximal T cells (71–74).

HER2-positive gastric cancer illustrates how targeted therapy and spatial immunity intersect. PD-L1 expression and immune microenvironment features can influence benefit from anti-HER2 plus immunotherapy strategies, and HER2-directed therapy can remodel immune-cell profiles (33, 34, 87). Spatial single-cell multi-omics also indicates that HER2 expression status is associated with microenvironmental heterogeneity (88). Thus, HER2 should be interpreted not only as a tumor-cell target but also as a context that may shape immune architecture.

The HER2 field is also moving from target positivity toward spatial and temporal heterogeneity. DESTINY-Gastric01 biomarker analyses show that HER2 expression and resistance biology remain clinically consequential after trastuzumab exposure, and translational work from DESTINY-Gastric06 links trastuzumab deruxtecan resistance to cellular and microenvironmental features (89, 90). Together with KEYNOTE-811 and HER2-associated spatial multi-omics, these data argue that HER2-positive tumors should be evaluated as evolving tumor-immune ecosystems rather than as a stable binary category (33, 87, 88, 91, 92).

CLDN18.2 provides another example. Multiplex immunohistochemistry has characterized the immune microenvironment and immunotherapeutic outcome in CLDN18.2-positive gastric cancer (93). These findings imply that CLDN18.2-targeted strategies may interact strongly with spatial immune context.

For CLDN18.2-positive disease, the pivotal SPOTLIGHT and GLOW trials, the earlier FAST trial, and ILUSTRO define the clinical target space, whereas multiplex IHC studies show that CLDN18.2 positivity is embedded in variable immune microenvironments (93–97). This creates an immediate spatial question: does a CLDN18.2-positive tumor contain accessible effector cells and antibody-compatible immune neighborhoods, or is the antigen located within an excluded, macrophage-rich, or poorly vascularized region?

HER2 and CLDN18.2 should be framed as spatially heterogeneous target-antigen niches rather than isolated eligibility labels. HER2-positive disease combines tumor-cell target expression with immune context, PD-L1 distribution, antibody-dependent immune mechanisms, and treatment-induced remodeling after trastuzumab-containing therapy. Trastuzumab deruxtecan biomarker and resistance analyses further suggest that post-ADC resistance may involve both residual target biology and microenvironmental escape (33, 34, 88–92, 98).

CLDN18.2-positive disease creates a parallel but distinct spatial problem. Zolbetuximab trials establish the clinical target, but CLDN18.2 expression can coexist with immune-excluded, macrophage-rich, or poorly vascularized regions, and CLDN18.2-directed T-cell engagement strategies make immune accessibility even more relevant (93–97). A stronger review should therefore ask not only whether HER2 or CLDN18.2 is present, but where target-positive tumor cells sit relative to effector cells, stromal barriers, vessels, and suppressive myeloid compartments.

TLS-focused therapeutic strategies remain attractive but early. If mature TLSs support local priming and PD-1 response, therapies that preserve or induce productive lymphoid niches could be valuable. However, TLS heterogeneity across primary and metastatic lesions means that induction of lymphoid aggregates alone may not be enough (10, 17, 22, 46, 47). The goal should be productive lymphoid organization, not simply more lymphocytes.

## 10 Discussion and future perspectives

Several barriers limit translation. First, many spatial studies are retrospective, single-center, and modest in sample size. Second, assays differ in resolution, chemistry, staining, segmentation, and computational definitions of neighborhood. Third, gastric cancer tissue sampling is difficult. Endoscopic biopsies may miss invasive-front, TLS-rich, stromal, or peritoneal niches, while surgical specimens may not reflect metastatic or post-treatment disease. Fourth, spatial phenotypes are dynamic. Fourth, Spatial immune niches should not be treated as fixed baseline labels. Treatment can remodel tumor-cell states, antigen availability, stromal architecture, vascular function, and immune-cell composition. Chemotherapy may increase antigen release and transiently enhance immune infiltration, but it may also select resistant subclones or leave residual lesions dominated by stromal and myeloid suppression. Anti-angiogenic therapy may improve immune entry during a window of vascular normalization, but adaptive hypoxia, perivascular trapping, or CAF/TAM compensation may later restore resistance (26, 42, 56, 82). HER2- or CLDN18.2-directed therapy can also alter target-antigen distribution and immune accessibility across residual tumor regions (88–90, 93).Longitudinal sampling is therefore central to spatial biomarker development. Baseline biopsies can identify the dominant pretreatment phenotype, but on-treatment and progression samples are needed to define niche transitions. A tumor may shift from stromal-excluded to inflamed after chemotherapy, from inflamed to myeloid-dominant after partial response, or from lymphoid-organized to metabolically suppressed during acquired resistance. Paired tissue, spatial imaging, ctDNA dynamics, and blood immune or myeloid markers could eventually support adaptive treatment assignment, although blood markers should complement rather than replace tissue context.

For trial design, acquired resistance should be analyzed as a spatial transition rather than as a simple loss of response. Future studies should report whether resistant lesions preserve effector-cell access, lose antigen presentation, gain CAF/ECM exclusion, accumulate suppressive TAMs, develop hypoxic or adenosine-rich regions, or emerge in a metastatic site with a distinct immune ecology. This would make spatial analysis useful not only for baseline selection, but also for explaining mixed progression and guiding second-line combinations.

Future trials should move from correlation to assignment. A stromal-excluded score could enrich patients for PD-1 plus stromal remodeling. A myeloid-dominant score could assign patients to PD-1 plus TAM reprogramming or myeloid checkpoint blockade. A lymphoid-organized inflamed score could identify patients likely to benefit from ICI-containing therapy without unnecessary additional toxicity. A vascular/metabolic score could guide anti-angiogenic combinations **Table 3**.

**Table 3:** Proposed implementation checklist for prospective spatial-niche-guided trials.

Decision question	Minimum spatial readout	Trial application	Key caveat
Is the tumor lymphoid-organized?	TLS maturity; CXCL13/B-cell pairing; tumor-proximal CD8 cells.	Enrich for ICI-sensitive disease or de-escalate broad combinations.	TLS presence alone is insufficient.
Is resistance stromal or myeloid?	CD8 exclusion distance; CAF/ECM score; SPP1/TREM2/VISTA/CLEVER-1 TAM neighborhoods.	Assign PD-1 backbone plus stromal, TAM, or myeloid-checkpoint module.	Broad stromal targeting may remove immune-supportive architecture.
Is the vascular niche reversible?	Hypoxia/VEGF features; perivascular immune trapping; plasma proteomic signal.	Select antiangiogenic plus ICI combinations and monitor adaptive escape.	Vascular normalization is transient and context-dependent.
Does target-positive disease have immune access?	HER2/CLDN18.2 IHC plus immune proximity, PD-L1 context, and post-treatment heterogeneity.	Pair targeted agents with immune-context selection and resistance biopsy.	Antigen positivity does not guarantee accessibility.
Is the dominant lesion metastatic?	Matched primary-metastasis profiling, especially peritoneal disease.	Use metastatic-site biology for assignment when feasible.	Endoscopic primary biopsy may miss the resistant niche.
Can resistance be reclassified?	Baseline plus on-treatment tissue/imaging, ctDNA, and blood immune/myeloid markers.	Reassign treatment at mixed progression or acquired resistance.	Blood markers should complement tissue context, not replace it.

ctDNA, circulating tumor DNA; ICI, immune checkpoint inhibitor; PD-L1, programmed death-ligand 1; VEGF, vascular endothelial growth factor.

TLS and digital pathology are good candidates for early prospective tests of this model. Mature TLS or lymphoid-organized scores could enrich for ICI-sensitive disease, whereas immature or suppressive lymphoid aggregates could trigger additional macrophage, stromal, or vascular modulation arms (23, 40, 41). The statistical endpoint should be treatment interaction rather than prognostic enrichment alone.

Metastatic-site profiling is a second priority. Peritoneal metastasis is common, difficult to sample, and biologically distinct; CAF-macrophage crosstalk and response-oriented immune-genomic features suggest that a primary-tumor spatial score may misclassify patients whose dominant resistant niche is metastatic (16, 48, 63). Multi-region sampling and matched primary-metastasis analysis should become standard in translational studies where feasible.

A third priority is integrating blood and tissue. The current library provides limited direct ctDNA/MRD evidence for this topic, so blood-based monitoring should be framed cautiously. Still, a future model could combine baseline spatial phenotype, circulating tumor DNA dynamics, systemic immune or myeloid biomarkers, and on-treatment tissue changes. This would allow resistance to be monitored as an evolving ecosystem rather than a one-time classification.

## 11 Conclusion

Gastric cancer immunotherapy resistance is not solely molecular; it is spatially organized. Standard biomarkers remain necessary, but they do not reveal whether effector cells contact malignant cells, TLSs are mature, CAF/ECM barriers exclude lymphocytes, macrophage programs dominate suppression, vascular dysfunction limits trafficking, or metastatic sites impose distinct immune ecologies.

Spatial immune niches provide a practical framework for integrating these dimensions into actionable categories. The field should now move from retrospective description to compact assays, prospective validation, and treatment-assignment trials that test whether niche-matched combinations improve outcomes beyond conventional biomarker selection.
